# Vitamin D Supplementation and Prior Oral Poliovirus Vaccination Decrease Odds of COVID-19 Outcomes among Adults Recently Inoculated with Inactivated Poliovirus Vaccine

**DOI:** 10.3390/vaccines12020121

**Published:** 2024-01-24

**Authors:** Brittany A. Comunale, Yea-Jen Hsu, Robin J. Larson, Aditi Singh, Erin Jackson-Ward, Lilly D. Engineer

**Affiliations:** 1 Department of Health Policy and Management, Johns Hopkins Bloomberg School of Public Health, Johns Hopkins University, Baltimore, MD 21205, USA; 2 Dartmouth-Hitchcock Medical Center, Geisel School of Medicine at Dartmouth, Hanover, NH 03755, USA; 3 Department of Palliative Medicine, Dartmouth-Hitchcock Medical Center, Lebanon, NH 03756, USA; 4 Department of Biological Sciences, University of California, San Diego, La Jolla, CA 92161, USA; 5 Cedars-Sinai Medical Center, Los Angeles, CA 90048, USA; 6 Department of Anesthesiology and Critical Care Medicine, Johns Hopkins School of Medicine, Baltimore, MD 21205, USA

**Keywords:** SARS-CoV-2 infection, COVID-19, poliovirus vaccines, OPV, IPV, social determinants of health, mucosal immunity, primary prevention

## Abstract

Background: Structural and functional commonalities between poliovirus and severe acute respiratory syndrome coronavirus 2 (SARS-CoV-2) suggest that poliovirus inoculation may induce antibodies that mitigate the coronavirus disease (COVID-19). No known studies have evaluated COVID-19 risk factors in adults recently vaccinated against poliovirus. Study Objective: Among adults with no history of COVID-19 infection or vaccination, who recently received an inactivated poliovirus vaccine (IPV), we sought to determine which biological factors and social determinants of health (SDOH) may be associated with (1) testing positive for SARS-CoV-2, (2) experiencing COVID-19 symptoms, and (3) a longer duration of COVID-19 symptoms. Methods: The influence of biological factors and SDOH on SARS-CoV-2 infection and COVID-19 symptoms were evaluated among 282 adults recently inoculated with IPV. Participant-reported surveys were analyzed over 12 months post-enrollment. Bivariate and multivariate linear and logistic regression models identified associations between variables and COVID-19 outcomes. Results: Adjusting for COVID-19 vaccinations, variants, and other SDOH, secondary analyses revealed that underlying conditions, employment, vitamin D, education, and the oral poliovirus vaccination (OPV) were associated with COVID-19 outcomes. The odds of testing positive for SARS-CoV-2 and experiencing symptoms were significantly reduced among participants who took vitamin D (OR 0.12 and OR 0.09, respectively). Unemployed or part-time working participants were 72% less likely to test positive compared with full-time workers. No prior dose of OPV was one of the strongest predictors of SARS-CoV-2 infection (OR 4.36) and COVID-19 symptoms (OR 6.95). Conclusions: Findings suggest that prophylactic measures and mucosal immunity may mitigate the risk and severity of COVID-19 outcomes. Larger-scale studies may inform future policies.

## 1. Introduction

When severe acute respiratory syndrome coronavirus 2 (SARS-CoV-2) (the virus that causes coronavirus disease 2019 (COVID-19)) first emerged in 2020, very little was known about who is most vulnerable, which risk factors heighten susceptibility, or how pathogenesis occurs [1]. In April 2020, a preliminary response to these questions was established when the World Health Organization stated that “older people, and those with underlying medical conditions (such as cardiovascular disease, chronic respiratory disease, or diabetes)” were more likely to experience severe disease [2]. Anecdotal evidence and real-time observations largely supported these initial claims, but over time, researchers have been able to collect ample data to demonstrate that other biological factors and social determinants of health (SDOH) are instrumental in influencing COVID-19–related health outcomes.

### 1.1. Age

In terms of specific biological factors, in March 2020, the Center for Disease Control and Prevention (CDC) was one of the first agencies to claim age as an indicative risk factor for developing a severe case of the COVID-19 disease [3]. Studies have since confirmed that adults older than 65 years account for 80% of COVID-19–related hospitalizations, and they can be up to 23 times more likely to die from COVID-19, compared with those aged 65 years and younger [4].

### 1.2. Underlying Medical Conditions

Aging is also associated with “inflammaging”, where constant exposure to inflammatory stimuli renders older adults more vulnerable to age-related diseases, which, in turn, can potentiate further inflammation [5]. Such conditions include cardiovascular disease, diabetes, and hypertension [6,7,8], all of which have been cited as common underlying medical conditions that are frequently associated with poor COVID-19 health outcomes [9,10,11,12,13].

### 1.3. Vitamin D

Vitamin D deficiency is another risk factor that may augment an individual’s susceptibility to COVID-19 [14]. A retrospective cohort study found that patients with a vitamin D deficiency were 77% more likely to test positive for SARS-CoV-2 compared with those who did not have this deficiency [15]. Since vitamin D is thought to have a protective effect against organs that may be impacted by severe COVID-19 disease, and it can disrupt pro-inflammatory processes associated with cytokine storms, it has been considered a potential agent in diminishing the likelihood of severe COVID-19 disease [16,17,18]. However, it should be noted that many studies examining vitamin D, that were published at the onset of the pandemic, were presented with several methodological limitations, and researchers have debated whether low vitamin D levels observed in COVID-19 patients were due to pre-existing, modifiable risk factors, or whether the levels represented an epiphenomenon of the disease as a reaction to the acute illness.

### 1.4. Biological Sex

As with other diseases, infections, and vaccines [19,20], sexual dimorphism in immune responses was observed during the COVID-19 pandemic. For instance, researchers have reported that women show greater antibody responses to COVID-19 vaccines [21,22], androgen steroid hormones may be associated with medical conditions that are more detrimental to health outcomes [23], and men have a higher risk of experiencing severe disease and dying from COVID-19 complications [24].

Although it is important to consider biological factors (i.e., age, biological sex), vitamin D deficiency, and underlying medical conditions in the context of SARS-CoV-2 infection and COVID-19 disease severity, these factors are not the only determinants of health. Social determinants of health (SDOH), which have been defined by the World Health Organization as “the conditions in which people are born, grow, work, live, and age, and the wider set of forces and systems shaping the conditions of daily life”, must also be considered [25]. Inequities related to social structures and systemic inequities may be one of the most critical “underlying conditions” to include in analyses that assess the impact of COVID-19 disease [26].

### 1.5. Education

Education is commonly considered a central social determinant of health due to the notion that those who venture into higher education are more likely to find prosperous employment opportunities, report better health perceptions, live in higher income and/or healthier neighborhoods, and are less likely to die from certain causes [27,28,29,30]. As it relates to COVID-19, education has been regarded as a potential protective factor against SARS-CoV-2 infection and COVID-19 disease severity. Individuals who have obtained an associate’s or bachelor’s degree are less likely to die from COVID-19 compared with those who have completed a lower level of education [12]. Such observed differences in health outcomes may be due, in part, to health literacy. Public health communications and risk mitigation methods that are presented at a higher reading level, or in a non-native language, will affect the degree to which that person may be able to read and understand messages that are intended to effectively protect the person and their family from SARS-CoV-2.

### 1.6. Economic Stability and Employment

Economic status may determine whether individuals have life-changing employment benefits that may include health insurance, preventive care services, paid sick leave, and other resources [31,32]. However, not all employment opportunities have been equitable in the context of the COVID-19 pandemic. The incidence rate of SARS-CoV-2 is higher in geographic areas that have higher historically marginalized populations, compared with areas where the median household income is higher. This disparity is likely due to increased workplace exposure, as lower-wage jobs disproportionately require face-to-face interactions, compared with jobs that have higher compensation [33,34,35]. Moreover, although telework opportunities burgeoned at the onset of the pandemic, such flexibility varies across industries (with hospitality, agriculture, and transportation services being less likely to offer remote work opportunities) [36,37]. The National Bureau of Economic Research found that nonremote workers are more likely to experience symptoms related to respiratory illness, with less protection from SARS-CoV-2, than individuals who had the ability to work remotely [38].

### 1.7. Race/Ethnicity

Inequities related to employment opportunities further exacerbate racial/ethnic disparities, as Black/African Americans and Hispanic/Latinos are more likely to be employed in high-risk industries, and less likely to have the opportunity to work from home [36,37,38,39,40]. These same structural systems increase the likelihood of worse COVID-19 outcomes, as Hispanic/Latinos, Asians, and particularly Black/African Americans are more often exposed to poor air quality and hazardous pollutants, intensifying the risk for asthma and other lung conditions, compared with whites [41,42,43]. Such continued exposure not only magnifies the chance of developing asthma or other lung conditions, but it also heightens the risk for COVID-19 complications and mortality [30,44].

Historically marginalized communities, which often have greater proportions of Asians, Black/African Americans, and Hispanic/Latinos, have both higher rates of COVID-19 and mortality compared with communities that predominantly comprise white populations and higher levels of income [45,46]. In terms of disease severity, Black/African Americans, Hispanic/Latinos, and American Indian or Alaska Natives were 2.9, 3.1, and 3.7 times more likely to be hospitalized for COVID-19, and 2.6, 3.9, and 7.2 times more likely to die from COVID-19 complications, compared with whites, respectively [47,48]. The disparate COVID-19 outcomes in specific communities can be attributed to structural and systemic access barriers to medical care, the incommensurate burden of underlying medical conditions, and higher levels of underinsurance [49]. Without access to health services, individuals tend to delay seeking treatment for COVID-19 until it is serious enough to require hospitalization [50]. Moreover, structurally disempowered populations, and those with an insecure documentation status, may not be able to take sick leave or feel comfortable accessing medical services to treat their SARS-CoV-2 infections [51,52].

### 1.8. Geographic Location

Populations in resource-deficient countries are more likely to be affected by viral outbreaks, enhanced contagions, and the emergence of new variants. One of the reasons why this difference exists is because there is inequitable access to COVID-19 vaccines in these areas compared with countries that have more resources [53]. However, most of the currently available COVID-19 vaccines were developed for the Alpha strain, and thus, they have provided less protection for subsequent variants that have emerged through antigenic drift and viral surface changes [54,55]. The most effective COVID-19 vaccines against circulating strains are the more recently produced bivalent boosters, which have been created to minimize the risk of severe SARS-CoV-2 from Omicron variants [56,57]. However, these boosters are only obtainable in fewer than forty countries, at time of writing.

Over the past three years, researchers have noted how complex and intertwined potential risk factors for COVID-19 truly are. As there is no single predictor, it is imperative that every research study accounts for as many primary factors as possible. Herein, a secondary data analysis of a single-center prospective clinical trial that evaluated immune responses and COVID-19 outcomes among participants who had recently received the inactivated poliovirus vaccine (IPV) is presented. The trial was initially created to assess whether a poliovirus vaccination could mitigate SARS-CoV-2 infection or COVID-19 symptoms due to structural and functional commonalities between the poliovirus and SARS-CoV-2. As the two viruses share a common protein that is responsible for viral replication (RNA-dependent RNA polymerase (RdRp)), it was theorized that inoculation against the poliovirus could create antibodies, including anti-RdRp, that could ultimately disrupt SARS-CoV-2 RdRp processes (i.e., viral replication) and diminish disease progression. Preliminary research suggests cross-reactivity would facilitate this process, in which a poliovirus antibody could recognize a SARS-CoV-2 antigen that is structurally similar to the poliovirus antigen, thus enabling potential protection against SARS-CoV-2 [58].

As such, participants in this study were given the poliovirus vaccine to test its theoretical protective effects against COVID-19. However, the type and extent of immunity induced by the poliovirus vaccination depends on the method of administration (i.e., the oral poliovirus vaccine (OPV) can induce innate or mucosal immunity, whereas an intramuscularly injected vaccine (IPV) induces a less diverse immunological response, solely through humoral immunity) [59]. Although OPV engenders a more robust immunological response, there is a risk of developing vaccine-derived poliovirus (VDPV) from the live, oral vaccine, OPV. Consequently, participants were given the inactivated version of the poliovirus vaccine, IPV, which poses no risk of VDPV.

Even though preliminary studies of this clinical trial have examined mounted antibodies and vaccination histories as potential predictors of immunological responses and health outcomes, a more holistic understanding of the study population has yet to be portrayed [60,61]. Thus, this secondary data analysis focuses on biological factors and social determinants of health as the main potential predictors of COVID-19 outcomes.

The aims of this study are to better understand the following questions: Among adults with no history of COVID-19 infection or vaccination who recently received an IPV, which biological factors and social determinants of health are associated with (1) testing positive for SARS-CoV-2, (2) experiencing COVID-19 symptoms, and (3) the duration of COVID-19 symptoms?

## 2. Materials and Methods

### 2.1. Clinical Trial Design

This study represents one part of a larger study, clinical trial NCT04639375, and it focuses on the retrospective, secondary data analysis of participant-reported COVID-19 outcome data that were collected during phone calls at 6 and 12 months after enrollment [60].

**Figure 1:**
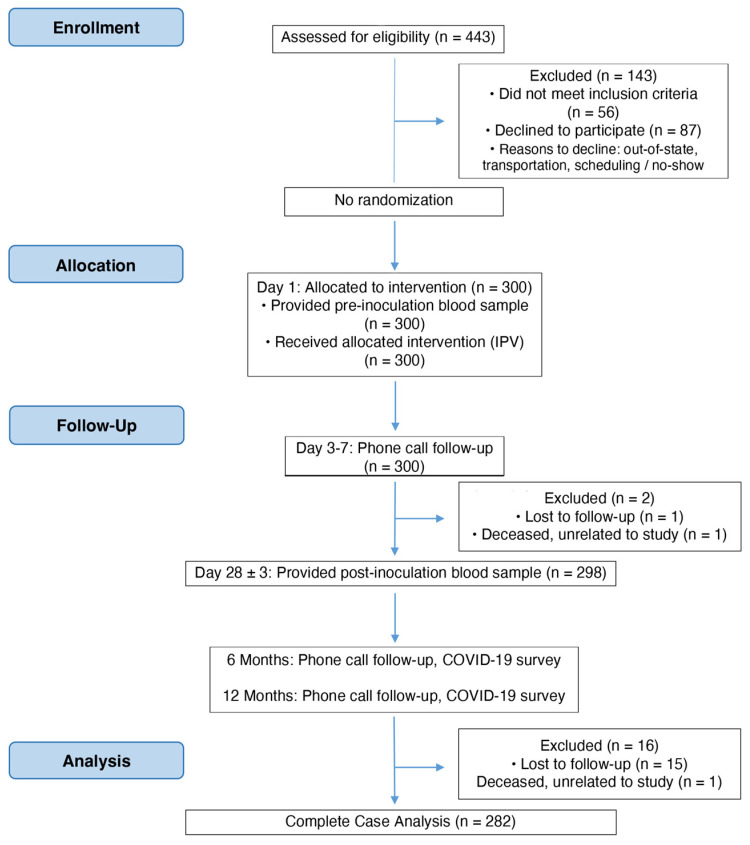
CONSORT flow diagram of clinical trial NCT04639375 and subsequent analyses.

The clinical trial was a single-arm, open-label, pilot study in which 300 individuals were given an IPV in Southern California between November 2020 and May 2021. Participants were enrolled in the study if they were between the ages of 18 and 80, with no active infectious disease, no previous history of COVID-19, no prior COVID-19 vaccinations, and no poliovirus vaccinations (IPV or OPV) over the past 12 years. Upon enrollment (Day 1), a baseline blood sample was drawn from participants, and a poliovirus injection (IPV) was given (Figure 1). The clinical site staff contacted participants via phone between Days 3 and 7 to record any adverse reactions to the injection. On Day 28 (+/− 3 days), participants returned to the clinic for a second blood draw. After that visit, participants were instructed to report any known SARS-CoV-2 exposures or positive test results as soon as possible via email or phone. From Day 28 onward, participants were also allowed to receive COVID-19 vaccinations, if they chose to do so.

At six and twelve months after the first clinic visit (Day 1), participants were contacted by clinical site staff via phone to record any known COVID-19 exposures or symptoms, as well as when and how the possible exposures took place. Sociodemographic information, including age, biological sex, race/ethnicity, employment status, completed education, and health insurance type, was also recorded. Participants were also asked about changes in current medications, including vitamin supplements, underlying medical conditions that were recorded at the first clinic visit, and vaccination histories. In the case of prior poliovirus vaccinations, the type of vaccine was also noted, as follows: OPV (sugar cube or liquid drop) or the intramuscularly injected vaccine, IPV. If participants received COVID-19 vaccinations after their second (Day 28) clinic visit, the brand (Pfizer-BioNTech (New York, NY, USA), Moderna (Cambridge, MA, USA), or Janssen (Johnson & Johnson, Titusville, NJ, USA)), dates of vaccination, and number of vaccinations in the series were recorded. Vaccination histories were verified by the clinical site staff using vaccination registry records and databases, as well as copies of vaccination cards provided by the participants, if available.

Per the clinical trial protocol, attempts were made to contact the participants up to three times via phone, and then by certified letter postal mail, for both 6- and 12-month follow-ups. The clinical trial coordinator noted the time and date of each attempt, and whether the participant was successfully reached. If all contact attempts were unsuccessful, the participant was considered “lost to follow-up”.

### 2.2. Data Source

The clinical trial from which analyzed data were derived was conducted in accordance with the International Conference on Harmonisation Good Clinical Practice (ICH GCP) and the United States Code of Federal Regulations (CFR), which is applicable to clinical studies (45 CFR Part 46, 21 CFR Part 50, 21 CFR Part 56, 21 CFR Part 312, and/or 21 CFR Part 812). Institutional review board approval (Advarra) was obtained for the study protocol, as were informed consent forms, recruitment materials, and all subject-facing materials. All participants provided written informed consent.

None of the authors were directly involved in data collection for clinical trial NCT04639375, but in the context of using these data as part of a larger study, the first and last authors obtained the necessary permissions from the clinical trial sponsor, EMO-Biotech, Inc., as well as permission from the Johns Hopkins School of Public Health Institutional Review Board.

### 2.3. Ethics

The Johns Hopkins School of Public Health Institutional Review Board reviewed this secondary analysis study and deemed it exempt.

### 2.4. Measures/Variables

#### 2.4.1. Independent Variables

A total of one continuous variable and fourteen categorical variables were selected a priori (Table 1).

Each of the independent variables were chosen based on the prior literature, with the exception of Dyslipidemia, Exposed to Omicron or Delta SARS-CoV-2 Strain, and Previously Received OPV. Dyslipidemia was included because it was a frequently noted underlying medical condition by the clinical trial site staff, and it is a well-known risk factor for cardiovascular disease [62,63]. The variable, Exposed to Omicron or Delta SARS-CoV-2 Strain, was included to see if there were differences between testing positive and experiencing symptoms after potential exposure to the disease during the 12 months after poliovirus inoculation. Some researchers have noted differences between the Delta and Omicron variants, with Omicron infections being less severe but more transmissible than those associated with the Delta strain [64,65,66]. The variable, Previously Received OPV, was included based on an earlier secondary data analysis, which explored the association between prior vaccinations, SARS-CoV-2 infection status, and COVID-19 outcomes between individuals in the same dataset in this study [61]. The analysis found that being in receipt of OPV was one of the strongest factors in influencing whether one tested positive for SARS-CoV-2 and/or experienced COVID-19 symptoms, indicating that receiving the OPV could be considered a contributing factor to health disparities and outcomes, as childhood vaccination guidelines vary based on where one lives (i.e., country).

All independent variables were recorded directly by the clinical site staff during the 6- and 12-month follow-ups conducted via telephone, with the exception of one independent variable that was generated by the primary author, as follows: Exposed to Omicron or Delta SARS-CoV-2 Strain. Responses for this variable were determined by comparing the SARS-CoV-2 exposure dates reported by participants, with daily SARS-CoV-2 variant strain reports presented on the “COVID-19 Variants in California” Dashboard on the official COVID-19 in California government website [67]. The dashboard displays which SARS-CoV-2 strains have been detected in California from January 1 2021 to present, based on the California Department of Health’s Genomic Surveillance Data and the California Department of Health’s communicable disease reporting and surveillance system, the California Reportable Disease Information Exchange.

#### 2.4.2. Dependent Variables

There were three dependent outcomes of interest, as follows: Participant Tested Positive for SARS-CoV-2 (Yes/No); Experienced COVID-19 Symptoms (Yes/No); and Days Experienced with COVID-19 Symptoms (continuous).

### 2.5. Missing Data

Eighteen participants (6.0%) did not return the 6- and 12-month phone calls or certified letters and were considered “lost to follow-up.” There were no systematic differences between the numbers or reasons for attrition. The median age and age range of those who discontinued the trial (51, 18–69 years) were similar to those of participants who continued participating in the study (55, 18–80 years). An equal number of males and females (50.0%) were lost to follow-up. To ensure changes in the linear or logistic regression models were due to new variables that were added to the model, and not a reduction in sample size from missing values, data from these 18 participants were removed to create a complete case analysis dataset. Given that the rate of attrition was close to the widely accepted 5% threshold, the impact of missing data is likely negligible.

### 2.6. Statistical Analysis

Statistical models and analyses were completed using RStudio (version 2022.12.0 + 353). Variables related to biological factors and social determinants of health were selected prior to analyses and based on the previous literature examining COVID-19 severity. Although age was analyzed as a continuous variable in this study, a categorical variable concerning age ranges (18–50; 51–64; 65–80) was also included and defined a priori, based on proposed epigenetic effects on immunity at particular ages, and prior research comparing groups in these age ranges [68,69,70,71,72].

Logistic regressions were performed for the two dichotomous variables of interest, Tested Positive for SARS-CoV-2 (Yes/No) and Experienced COVID-19 Symptoms (Yes/No); linear regressions were conducted for the continuous variable of interest, Days Experienced with COVID-19 Symptoms.

Given that there were several primary variables of interest for this study, bivariate linear and logistic regression models were constructed to assess the effects of each independent variable on the respective dependent outcome. The main effects of the interaction terms that were related to the research questions were also tested.

To address the hypothesis that biological and social determinants of health may potentially contribute to testing positive for SARS-CoV-2 (Aim 1), multivariate logistic regressions were performed using forward selection through a phased approach. The first model was a bivariate regression of Education Completed and Testing Positive for SARS-CoV-2, due to lower educational attainment being associated with lower wage compensation and jobs that require face-to-face interactions, increasing the likelihood of contracting SARS-CoV-2. The second model was a bivariate regression of Race/Ethnicity and Testing Positive for SARS-CoV-2, given the disproportionate levels of Hispanic/Latino individuals contracting COVID-19, which is likely due to the higher risk of exposure at work compared with white individuals, or living in multi-generational homes [73]. Various combinations and iterations of the remaining independent variables were then included in subsequent models. Changes in odds ratios, standard error, and *p* values were analyzed at each step to see if additional variables may confound the primary relationship. Additionally, the Akaike information criterion (AIC) and Bayesian information criterion (BIC) were calculated at each step to further analyze the model fit. Models with smaller AIC and BIC values indicated which may be more parsimonious and which accurately fit the data. Of these, the model with the lowest value was selected as the best fit. The likelihood ratio and Wald tests were also used to see if the regression coefficients for the predictor variables and interaction terms, which were included in the final model, statistically improved the overall fit; models were compared with and without the variable of interest. The odds ratios and 95% confidence intervals were reported for the final model. *p* values less than 0.05 were considered statistically significant.

The same methodological process was repeated for the second dichotomous outcome to test the hypothesis that biological factors and social determinants of health may potentially contribute to experiencing COVID-19 symptoms (Aim 2). The first logistic regression model was a bivariate regression of Underlying Medical Conditions and Experiencing COVID-19 Symptoms, which was chosen by considering how underlying medical conditions are not only impacted by both genetic and socio-behavioral factors, but also understood to be associated with a more severe version of the COVID-19 disease [9,10,13]. The second model was a bivariate regression of Age (categories) and Experiencing COVID-19 Symptoms, since age is related to COVID-19 severity, leading to the assumption that those with suboptimal immune systems due to age may be more likely to experience symptoms and less likely to be asymptomatic. Again, various combinations and iterations of the remaining independent variables were then included in subsequent models. Changes in odds ratios, the standard error, and *p* values were analyzed at each step to see if additional variables may confound the primary relationship. AIC, BIC, the likelihood ratio, and Wald tests were also performed. Odds ratios (adjusted and unadjusted) and 95% confidence intervals were reported for the final model. *p* values less than 0.05 were considered statistically significant.

To address the hypothesis that the duration of symptoms, an indicator of severity, may be affected by biological and social determinants of health (Aim 3), multivariate linear regressions were performed using forward selection. The first model was a bivariate linear regression of Underlying Medical Conditions and Days Experienced with COVID-19 Symptoms, given that prior research, and recommendations from the CDC and WHO, noted how individuals with underlying medical conditions are more likely to experience severe COVID-19. The second model was a bivariate linear regression of Received COVID-19 Vaccine and Days Experienced with COVID-19 Symptoms, as anecdotal and empirical data suggest that the COVID-19 vaccines minimize the likelihood of experiencing severe COVID-19. Again, using a phased approach, independent variables were either added or subtracted one at a time in subsequent models. Changes in regression coefficients, the standard error, and *p* values were analyzed during each step to see if additional variables may confound the primary relationship. AIC, BIC, the likelihood ratio, and Wald tests were also performed. Regression coefficients and 95% confidence intervals were reported for the final model. *p* values less than 0.05 were considered statistically significant.

Two independent variables, Education Completed and Race/Ethnicity, were re-leveled after bivariate analyses showed no significant differences across categories or insufficient sample sizes for a particular category. As a result, to simplify analyses and reduce the number of small cells, similar categories were collapsed (i.e., for the variable Education Completed, “Master’s Degree” and “Doctoral Degree”, responses were combined as “Graduate Degrees”; for the variable Race/Ethnicity, participants who identified as Asian, Black/African American, or Other were combined due to the insufficient sample size, compared with their white and Hispanic/Latinx counterparts).

## 3. Results

### 3.1. Demographics and Clinical Characteristics

A total of 282 participants were included in the complete case analysis. Supplementary Table S1 summarizes the demographic and clinical characteristics of the analyzed study population.

### 3.2. Main Effects of Potential Contributory Factors

#### 3.2.1. Bivariate Analysis

Supplementary Table S2 summarizes the main bivariate effects of each independent variable on the three outcomes of interest, as follows: Participant Tested Positive for SARS-CoV-2 (Yes/No); Experienced COVID-19 Symptoms (Yes/No); and Days Experienced with COVID-19 Symptoms (continuous). Without adjusting for other factors, bivariate analyses suggest that eight individual predictors could influence whether one tests positive for SARS-CoV-2 (Employment Status, Underlying Medical Conditions, Diabetes, Hypertension, Vitamin D Supplementation, Received COVID-19 Vaccine, Exposed to Omicron or Delta Strain, and Previously Received OPV); nine predictors could influence whether one experiences symptoms related to COVID-19 (Employment Status, Underlying Medical Conditions, Diabetes, Hypertension, Dyslipidemia, Vitamin D Supplementation, Received COVID-19 Vaccine, Exposed to Omicron or Delta Strain, and Previously Received OPV); and five predictors could influence the length of time that one may experience COVID-19 symptoms (Hypertension, Vitamin D Supplementation, Received COVID-19 Vaccine, Exposed to Omicron or Delta Strain, and Previously Received OPV).

Additionally, only one interaction term, Vitamin D Supplementation × Education Completed, was statistically significant in influencing two outcomes of interest, as follows: Tested Positive for SARS-CoV-2 and Experienced COVID-19 Symptoms.

Although preliminary analyses suggest that one’s race/ethnicity could influence COVID-19 infection status and symptoms, the only race/ethnicity that demonstrated a significant difference from the reference category (white) was Asian, and this subpopulation comprised only 9.2% of the analyzed cohort, with an insufficient sample size of 26 people. Therefore, there were no significant differences across race/ethnicities in this study population, though additional investigations of this variable with larger populations are recommended.

To more accurately understand possible predictors, parsimonious logistic and linear regression models, adjusted for other instrumental factors, were analyzed.

#### 3.2.2. Final Logistic Regression Models for Dichotomous Outcomes of Interest: Tested Positive for SARS-CoV-2 (Aim 1) and Experienced COVID-19 Symptoms (Aim 2)

A phased approach for multivariate regressions using forward selection revealed that the most parsimonious model of factors that influenced testing positive for SARS-CoV-2, or experiencing COVID-19 symptoms, included Underlying Medical Conditions, Employment Status, Vitamin D Supplementation, Education Completed, Received COVID-19 Vaccine, Exposed to Omicron or Delta Strain, Previously Received OPV, and the interaction between Vitamin D Supplementation and Education Completed (Table 2).

Participants who had underlying medical conditions, including but not limited to diabetes, hypertension, dyslipidemia, asthma, and cardiac conditions, were 2.36 times more likely to test positive for SARS-CoV-2 (*p* < 0.01) and 4.5 times more likely to experience COVID-19 symptoms (*p* < 0.001) compared with participants who did not have underlying medical conditions.

Employment status significantly influenced whether one tested positive or experienced symptoms, likely due to workplace exposure and social interactions. Those who worked part-time and those who were not employed were 72% (*p* < 0.01) and 71% (*p* < 0.001) less likely to test positive, respectively, compared with those who worked full-time. Moreover, these subpopulations were 78% (*p* < 0.01) and 73% (*p* < 0.001) less likely to experience COVID-19 symptoms compared with those working full-time, respectively. These individuals spent most of their time at home, and thus, they were more likely to be isolated away from potential COVID-19 exposures, compared with those who were employed full-time.

Level of education completed also had an effect on whether one tested positive for SARS-CoV-2, though there was no comparable effect on whether someone experienced symptoms. Participants who had completed graduate school were 70% less likely to test positive (*p* < 0.05), compared with participants who had earned an Associate’s or Bachelor’s degree as their highest level of education.

The most influential factors that affected SARS-CoV-2 infection status, and whether someone experienced COVID-19 symptoms, were previously receiving OPV and taking vitamin D supplements on a regular basis. Participants who reported never receiving OPV, either as a child or for travel purposes, instead receiving only IPV, were 4.36 times more likely to test positive for SARS-CoV-2 (*p* < 0.001) and 6.95 times more likely to experience COVID-19 symptoms (*p* < 0.001) compared with those who had received OPV at some point in their lives. Similarly, those who took vitamin D supplements on a regular basis were 88% less likely to test positive (*p* < 0.001) and 91% less likely to experience COVID-19 symptoms (*p* < 0.001) compared with those who did not take vitamin D supplements.

As the interaction between Vitamin D Supplementation (Yes) × Education Completed (Graduate Degree) revealed an odds ratio greater than one (8.10 and 8.06, respectively), the association was stronger than expected. Considering this multiplicative effect, participants who took vitamin D supplements and held a graduate degree were 70.8% less likely to test positive for SARS-CoV-2 (*p* < 0.05) and 69.5% less likely to experience COVID-19 symptoms (*p* < 0.05) compared with participants who held an Associate’s or Bachelor’s degrees who did not take vitamin D supplements.

Although models for both SARS-CoV-2 infection and COVID-19 symptoms noted whether participants had received at least one COVID-19 vaccine (Pfizer-BioNTech, Moderna, or Johnson & Johnson) or not, this factor was not statistically significant in either model.

The SARS-CoV-2 variant strain that one may have been exposed to, either Delta or Omicron, significantly influenced whether one experienced symptoms. Participants who were exposed to the Omicron strain were 73% less likely to experience symptoms (*p* < 0.001) compared with those who had been exposed to the Delta strain. Whether one was exposed to the Delta or Omicron strain also had a significant effect (*p* = 0.05) on testing positive.

#### 3.2.3. Final Linear Regression Model for Continuous Outcome of Interest: Days Experienced with COVID-19 Symptoms (Aim 3)

The final linear regression model for the continuous outcome of interest, Days Experienced with COVID-19 Symptoms, included five variables, as follows: Hypertension, Vitamin D Supplementation, Received COVID-19 Vaccine, Exposed to Omicron or Delta Strain, and Previously Received OPV (Table 3).

Two factors significantly affected the duration of COVID-19 symptoms, as follows: taking vitamin D supplements and previously receiving OPV. Participants who took vitamin D supplements on a regular basis experienced symptoms for 3.45 fewer days compared with those who did not take vitamin D supplements. Additionally, participants who had never received OPV experienced symptoms for 5.81 more days on average than those who had previously received OPV at some point in their life.

## 4. Discussion

### 4.1. Synthesis

The secondary data analysis demonstrated which of the pre-selected, biological factors and social determinants of health had an influence on participants’ COVID-19 health outcomes, and it was found that the extent of this impact may depend on whether participants had previously received OPV.

Adjusting for underlying medical conditions, employment status, educational attainment, receiving the COVID-19 vaccination, and exposure to Omicron or Delta variants, analyses revealed that vitamin D supplementation and receiving OPV each independently contributed to all three COVID-19 outcomes, as follows: whether individuals tested positive for SARS-CoV-2, experienced COVID-19 symptoms, and length of time that they experienced those symptoms.

Although vitamin D serum levels were not tested in the clinical trial, it was assumed the participants who regularly took vitamin D supplements did not have vitamin D deficiencies at the time of the study. Prior research shows that people who regularly take vitamin D supplements or have a more vitamin D-rich diet have a lower risk of vitamin D deficiency [74,75]. Moreover, several studies have noted that obtaining vitamin D solely through sunlight or diet is not usually sufficient, and vitamin D supplementation is critical for achieving and maintaining high enough levels [76,77]. As such, if the participants who took vitamin D supplements were re-framed as individuals who likely did not have vitamin D deficiencies, then the data would replicate previous findings showing that vitamin D may provide a protective effect against COVID-19, compared with those who have a vitamin D deficiency [14,16,18,78]. Furthermore, recently published data exploring the association between vitamin D deficiency and the COVID-19 vaccination demonstrate that vitamin D-deficient individuals are at risk of exhibiting poor immunological responses to COVID-19 vaccinations. Di Filippo et al. suggest elevating vitamin D levels in vulnerable populations, prior to administering COVID-19 vaccinations, to boost the immunological response and to foster long-term protection against the virus [79]. Such findings may support the notion that vitamin D supplementation has a potential bolstering effect on OPV, reinforcing the idea that it provides enhanced protection against the SARS-CoV-2 infection and COVID-19 outcomes in this study. Given that socioeconomic factors are known to have an impact on the consumption of vitamins [80], that low vitamin D levels are prominent in resource-deficient countries [81], and that recommendations for vitamin D supplements often require a medical provider’s intervention, access to vitamin D supplementation can be considered a social determinant of health.

The other contributing factor that influenced all three COVID-19 outcomes was whether participants had previously received OPV. Participants who had not previously received OPV were more likely to test positive or have symptoms, and they were more likely to experience those symptoms for longer.

These findings indicate that previously receiving OPV may provide a layer of protection against the SARS-CoV-2 infection and COVID-19 symptoms in individuals who have recently received IPV, because their immune systems would be mucosally primed. If the immunological responses mirrored the IgA responses (antibodies found in mucosal secretions) observed by Herremans et al. (1999), then, at the time when participants received IPV in the clinical trial, a mucosal immune response would have been initiated [82]. A high level of IgA antibodies would be particularly beneficial in combatting COVID-19, as serum IgA has been noted to be more powerful in neutralizing the SARS-CoV-2 virus compared with other antibody types [83]. Furthermore, when comparing with results from the 2014 study by John et al., participants who had never received OPV displayed less robust immunological responses compared with those who had received OPV, because they did not have the opportunity to obtain mucosal immunity before receiving IPV during the first visit in the clinical trial [84]. A possible explanation as to why poliovirus immunity might provide a protective effect against SARS-CoV-2 infection and COVID-19 outcomes may be that the poliovirus vaccinations induced RdRp antibodies in the study participants, and SARS-CoV-2 viral replication may have been inhibited, as shown previously in vitro [58]. Serological data of both IgA responses and RdRp inhibition activities are needed to confirm these hypotheses.

Several other social determinants of health were included in the final regression models, such as underlying medical conditions and employment status, which also contributed to COVID-19 health outcomes. Participants who had underlying medical conditions were not only more likely to test positive, but they were also more likely to experience COVID-19 symptoms compared with those who did not have underlying medical conditions. The existing literature suggests that people with such conditions are more likely to experience severe COVID-19 cases compared with healthier individuals, but the evidence does not indicate that this subpopulation would be more likely to test positive. A possible explanation for this finding is that there may be more positive COVID-19 cases among individuals who do not have underlying conditions, but if they are asymptomatic, they may not be testing themselves. In contrast, as the prior literature suggests, and as this study further validates, those with underlying medical conditions are more likely to experience symptoms, and therefore, they may be more likely to test and obtain a positive diagnosis.

Employment status played a role in both testing positive and experiencing symptoms. Both part-time employees and those who were not employed were less likely to test positive or experience symptoms compared with participants who had earned their Bachelor’s or Associate’s degrees. These findings aligned with our hypotheses, which were based on the assumption that people who are employed full-time may be exposed to the virus at work for longer periods of time. However, the survey responses related to employment status did not specify whether employees were working remotely or in-person, so such explanations cannot yet be confirmed, and should be noted in future studies.

Although the study hypothesized that there is an association between educational attainment and COVID-19 outcomes, we expected those who had completed lower levels of education might increase the likelihood of testing positive due to associations with lower wage compensation and face-to-face interactions in the workplace [33,34,35]. The educational category of completing High School or Lower did not produce any significant associations in the final regression models. However, we found that graduate degree holders were less likely to test positive, and this effect was augmented due to the interaction between vitamin D supplementation and level of education completed.

Even though many interaction terms were tested based on hypotheses concerning one biological or social factor influencing another, only one interaction was statistically significant and was found in both the Tested Positive for SARS-CoV-2 and Experienced COVID-19 Symptoms models, as follows: Vitamin D Supplementation × Education Completed, specifically those holding graduate degrees. The relationship between testing positive for SARS-CoV-2 or experiencing COVID-19 symptoms and taking vitamin D supplements warrants further investigation. It is possible that the relationship may be influenced by a third variable, such as higher education, for a variety of reasons. It is possible that participants with graduate degrees are more likely to read scientific articles related to COVID-19 risk mitigation measures and/or those that discuss vitamin D deficiency in the context of the COVID-19 pandemic adapt their everyday behaviors accordingly. Alternatively, participants with graduate degrees may be more likely to have prosperous employment opportunities, which may not only affect occupational exposure (i.e., more likely to work in a more isolated space, such as a laboratory or individual office; therefore, less exposed to potentially infected individuals), but also increase the likelihood of having employment benefits, such as health insurance, which may encourage access to preventive medical care and health service utilization. Although these specific conjectures should be validated in future research, other studies have noted that use of vitamin supplementation has increased during the COVID-19 pandemic [85,86], and higher education may also be associated with taking vitamin supplements [87,88].

### 4.2. Strengths and Limitations

The original study design possesses several strengths and limitations that affected the secondary data analyses. The study population demographics show that 48% of the participants had underlying medical conditions. Although this balanced proportion is helpful when comparing two groups in statistical analyses, there is concern that such a high number of participants living with underlying conditions, which may increase the likelihood for severe COVID-19 on its own, could skew the data in a way that does not align with other experimental studies that typically recruit healthy volunteers. However, including participants who are more representative of the U.S. population enhances the study’s generalizability.

The study’s sample size, single-arm design, and absence of serological data at the 6- and 12-month follow-ups should be considered critical limitations. Utilizing a complete-case analysis further reduced the sample size, though the 6% attrition rate’s close proximity to the widely accepted 5% threshold suggests that the impact of missing data is likely negligible. The single-arm study design was intentional, as the trial was intended to pilot a proof-of-concept study to assess poliovirus vaccinations in the context of the COVID-19 pandemic. Future studies should further explore the potential contributors to COVID-19 outcomes presented herein, such as dosage, timing, and formulations of vitamin supplementations. Additionally, other COVID-19 risk factors, such as smoking, should be explored in a multicenter, with a larger study population, and objective measures, in order to more effectively confirm hypotheses and validate data acquired through more subjective means. As the survey data, analyzed herein, originated from the 6- and 12-month follow-ups, recall and survivor biases must also be considered.

Although participants were contacted 6 and 12 months after their first clinic visit, in the hope of reducing the time spent remembering possible SARS-CoV-2 exposures and dates, recall bias may have occurred. However, the clinical trial site staff did instruct participants to contact them via email or phone as soon as SARS-CoV-2 exposures or positive test results became known; then, these intermittent reports were confirmed at the 6- and 12-month phone calls. Survivor bias is highly unlikely, because two of the 300 participants (0.67%) passed away during the study, both due to causes unrelated to COVID-19.

## 5. Conclusions and Implications for Research and Practice

Although the emergence of the novel coronavirus disease in 2019 raised more questions than researchers had answers, data about potential risk factors that could influence COVID-19 outcomes have become increasingly available. This secondary data analysis examined the effects of 15 independent variables on three COVID-19 outcomes, as follows: testing positive for SARS-CoV-2, experiencing COVID-19 symptoms, and the length of time that COVID-19 symptoms were experienced. The resulting models highlighted the importance of considering several biological factors and social determinants of health due to the intertwined nature of these factors. Two elements that were found to minimize the risks of SARS-CoV-2 infection and COVID-19 severity were regularly taking vitamin D supplements and previously receiving OPV; two factors that were found to heighten the risks were underlying medical conditions and full-time employment.

If larger studies further confirm the preliminary associations indicated above, public health policies have the opportunity to protect more vulnerable populations and equitably distribute resources that may minimize the impact of the COVID-19 disease and its severity. For example, policies that encourage shared decision-making, concerning the regular use of vitamin D supplementation, and that support the expansion of vitamin accessibility, could have profound implications. Not only are historically marginalized populations inequitably affected by upstream health factors, such as structural and systemic access barriers to preventive medical care, but they are also disproportionately affected by the downstream effects of more severe versions of the COVID-19 disease. Implementing such policies could be a cost-effective and minimally invasive way to positively bolster vulnerable populations in resource-deficient areas.

Similarly, policies that encourage OPV vaccination for those who have never received OPV could provide protective effects through mucosal immunity in populations that are more susceptible to COVID-19 disease and who might develop a more severe version of the disease. All recommendations should be informed by future, larger-scale studies that investigate vaccination schedules (i.e., number and frequency of vaccinations), administration methods (i.e., IPV, OPV, or a combination of the two), the effects of OPV mucosal priming on new SARS-CoV-2 variants, and how each variable may also be influenced by other biological factors and social determinants of health. Policy recommendations for vaccination schedules must be personalized in a way that accounts for those factors.

Given that this study validated the prior literature and anecdotal evidence suggesting that the presence of underlying medical conditions strongly predicts COVID-19 outcomes, public health agencies should continue to recommend cautious guidelines for more vulnerable individuals. In the workplace, protective measures should be encouraged for employees with underlying medical conditions, as well as for those who work full-time and may be at risk of higher SARS-CoV-2 exposure in the workplace. Corporations and organizations that operate in more historically marginalized and resource-deficient areas should be prioritized in terms of public health education and targeted messaging, since those communities are in greater danger of contracting the SARS-CoV-2 infection and severe COVID-19 disease, and are less likely to be afforded the privilege of taking time off of work to address medical concerns. Recommended protective measures could include increased flexibility (in terms of being able to perform work activities remotely), paid time off to utilize medical services and address health concerns, the provision of sanitary resources, company-provided SARS-CoV-2 infection testing, the relocation of vulnerable employees to more protected workplace areas, and/or mandatory mask mandates for more densely populated organizational events or locations. Providing more resources and flexible work options for individuals who are more susceptible to SARS-CoV-2 infection will not only promote more equitable and safe workplace environments, but it will also embolden other organizations to champion the needs of their employees.

As demonstrated in this study, recognizing who may be most vulnerable, and which risk factors may heighten susceptibility, is critical in formulating public health policies that may dictate resource allocation to achieve optimal health outcomes. If more prophylactic measures, such as the administration of vitamin D supplements or OPV, are encouraged, and additional risk mitigation measures are implemented for more vulnerable populations (i.e., those with underlying health conditions, those who are employed full-time, etc.), the burden of severe COVID-19 cases on hospital services and public health infrastructure may be alleviated, and there may be a subsequent reduction in national SARS-CoV-2 infection rates.

## Figures and Tables

**Table 1:** Independent Variables.

Variable	Factor Type	Variable Type	Levels
Age	Biological	Continuous	N/A (continuous, 18–80)
Age (categories)	Biological	Ordinal	3 (18–50; 51–64; 65–80)
Biological Sex	Biological	Dichotomous	2 (Male; Female)
Education Completed	SDOH	Ordinal	4 (High School or Less; Bachelor’s or Associate’s; Master’s Degree; Doctoral Degree)
Employment Status	SDOH	Ordinal	3 (Not Employed; Part-Time; Full-Time)
Previously Received OPV	SDOH	Dichotomous	2 (Yes; No)
Received COVID-19 Vaccine	SDOH	Dichotomous	2 (Yes; No)
Currently Taking Vitamin D Supplementation	SDOH	Dichotomous	2 (Yes; No)
Underlying Medical Conditions	SDOH	Dichotomous	2 (Yes; No)
Diabetes	SDOH	Dichotomous	2 (Yes; No)
Hypertension	SDOH	Dichotomous	2 (Yes; No)
Dyslipidemia	SDOH	Dichotomous	2 (Yes; No)
Race/Ethnicity	SDOH	Categorical	4 (white; Hispanic/Latinx; Asian; Other)
Health Insurance Types	SDOH	Categorical	4 (Private; Medicare; Medicaid; No Insurance)
Exposed to Omicron or Delta SARS-CoV-2 Strain	Other-Potential Confounder	Dichotomous	2 (Delta; Omicron)

**Table 2:** Final Logistic Regression Models for Dichotomous Outcomes: ‘Tested Positive for SARS-CoV-2’ and ‘Experienced COVID-19 Symptoms’.

Independent Variable	Adjusted Odds Ratio	95% Confidence Interval	*p*-Value	Adjusted Odds Ratio	95% Confidence Interval	*p*-Value
**Underlying Medical Conditions**						
No	1.0	---	---	1.0	---	---
Yes	**2.36**	**1.24–4.57**	**<0.01**	**4.50**	**2.25–9.33**	**<0.001**
**Employment Status**						
Full-Time	1.0	---	---	1.0	---	---
Not Employed	**0.29**	**0.15–0.58**	**<0.001**	**0.27**	**0.13–0.56**	**<0.001**
Part-Time	**0.28**	**0.11–0.69**	**<0.01**	**0.22**	**0.08–0.58**	**<0.01**
**Vitamin D Supplementation**						
No	1.0	---	---	1.0	---	---
Yes	**0.12**	**0.03–0.39**	**<0.001**	**0.09**	**0.02–0.31**	**<0.001**
**Education Completed**						
Bachelor’s/Associate’s Degree	1.0	---	---	1.0	---	---
Graduate Degree	**0.30**	**0.10–0.83**	**<0.05**	0.42	0.14–1.20	0.11
High school or less	0.86	0.41–1.79	0.69	0.67	0.30–1.47	0.32
**Received COVID-19 Vaccine**						
No	1.0	---	---	1.0	---	---
Yes	0.56	0.30–1.04	0.07	0.57	0.30–1.09	0.09
**Exposed to Omicron or Delta Strain**						
Delta	1.0	---	---	1.0	---	---
Omicron	0.55	0.30–1.01	0.05	**0.27**	**0.14–0.52**	**<0.001**
**Previously Received Oral Polio Vaccine (OPV)**						
Yes	1.0	---	---	1.0	---	---
No	**4.36**	**2.23–8.79**	**<0.001**	**6.95**	**3.25–15.83**	**<0.001**
**Vitamin D × Education Completed** (Yes × Graduate Degree)	**8.10**	**1.13–60.40**	**<0.05**	**8.06**	**1.10–63.75**	**<0.05**
**Vitamin D × Education Completed** (Yes × High School or Less)	1.12	0.19–6.52	0.90	0.77	0.10–5.43	0.80

**Table 3:** Final Linear Regression Model for Continuous Outcome: ‘Days Experienced with COVID-19 Symptoms’.

Independent Variable	Regression Coefficient (*ß*)	95% Confidence Interval	*p*-Value
**Hypertension** (Yes)	1.99	−0.29–4.27	0.08
**Vitamin D Supplementation** (Yes)	**−3.45**	**−5.81–−1.09**	**<0.01**
**Received COVID-19 Vaccine** (Yes)	−2.17	−4.35–0.03	0.06
**Exposed to Omicron or Delta Strain** (Omicron)	−2.04	−4.24–0.16	0.07
**Previously Received Oral Polio Vaccine** (No)	**5.81**	**3.35–8.28**	**<0.001**

## Data Availability

The datasets analyzed for this study may be available upon request.

## Supplementary Materials

The following supporting information can be downloaded at: https://www.mdpi.com/article/10.3390/vaccines12020121/s1, Table S1. Participant Demographics and Characteristics. Table S2. Main Effects of Predictor Variables.
