# Supplementary material for: Vitamin D Supplementation and Prior Oral Poliovirus Vaccination Decrease Odds of COVID-19 Outcomes among Adults Recently Inoculated with Inactivated Poliovirus Vaccine
Source: Vaccines (Basel). 2024 Jan 24;12(2):121. doi: 10.3390/vaccines12020121 (PMC10892023; doi:10.3390/vaccines12020121)
Supplement: Supplementary file 1 [file vaccines-12-00121-s001.zip › vaccines-2822045-supplementary.pdf]

Supplementary Material

11. Supplementary Figures and Tables

11.1 **Supplementary Table S1.** Participant Demographics and Characteristics

**Characteristic**	**Frequency (%) (N = 282)**	**Characteristic**	**Frequency (%) (N = 282)**
**Age (years)**		**Underlying Medical Conditions**	
Mean	51.4	*Yes*	135 (47.9)
Median	55.0	*No*	147 (52.1)
Range	18-80	**Hypertension**	
**Age (years by category)**		*Yes*	91 (32.3)
18-50	123 (43.6)	*No*	191 (67.7)
51-64	86 (30.5)	**Diabetes**	
65-80	73 (25.9)	*Yes*	58 (20.6)
**Biological Sex**		*No*	224 (79.4)
Male	130 (46.1)	**Dyslipidemia**	
Female	152 (53.9)	*Yes*	51 (18.1)
**Race/Ethnicity**		*No*	231 (81.9)

White	173 (61.3)	**Received COVID-19 Vaccine**	
Hispanic/Latinx	67 (23.8)	*Yes*	150 (53.2)
Asian	26 (9.2)	*No*	132 (46.8)
Other	16 (5.7)	**Vitamin D Supplementation**	
**Employment Status**		*Yes*	100 (35.5)
Full-Time	121 (42.9)	*No*	182 (64.5)
Part-Time	44 (15.6)	**Previously Received Oral Polio Vaccine (OPV)**	
Not Employed	117 (41.5)	*Yes*	207 (73.4)
**Education Completed**		*No*	75 (26.6)
Graduate Degree	50 (17.7)	**Tested Positive for SARS-CoV-2**	
Bachelor or Associate	106 (37.6)	*Yes*	99 (35.1)
High School or Less	126 (44.7)	*No*	183 (64.9)
**Exposed to Omicron or Delta SARS-CoV-2 Strain**		**Experienced COVID-19 Symptoms**	
Omicron	160 (56.7)	*Yes*	111 (39.4)
Delta	122 (43.3)	*No*	171 (60.6)

Health Insurance		Duration of COVID-19 Symptoms	
Private	157 (55.7)	*Mean*	10.3 days
Medicare	70 (24.8)	*Median*	7.0 days
Medicaid	31 (11.0)	*Range*	1 day - 120 days (N=1)
No Insurance	24 (8.5)		

11.2 **Supplementary Table S2.** Main Effects of Predictor Variables

	Tested Positive for SARS-CoV-2 (Yes/No)			Experienced COVID-19 Symptoms (Yes/No)			Days Experienced COVID-19 Symptoms		
**Indep. Variable**	**Odds Ratio (Unadj)**	**95% CI**	**p-Value**	**Odds Ratio (Unadj)**	**95% CI**	**p-Value**	***ß Coefficient***	**95% CI**	**p-Value**
**Age (years by category)**									
18-50	1.0	---	---	1.0	---	---	---	---	---
51-64	0.93	0.52 – 1.65	0.801	0.74	0.42 – 1.31	0.307	-0.93	-3.66 – 1.80	0.502
65-80	0.85	0.46 – 1.56	0.599	0.78	0.43 – 1.40	0.401	0.89	-1.98 – 3.76	0.541
**Biological Sex**									
Female	1.0	---	---	1.0	---	---	---	---	---

Male	1.16	0.71 – 1.89	0.555	1.05	0.65 – 1.70	0.839	-0.16	-2.48 – 2.1	0.894
**Race/Ethnicity**									
white	1.0	---	---	1.0	---	---	---	---	---
Hispanic/ Latinx	0.86	0.48 – 1.54	0.618	0.88	0.49 – 1.56	0.668	-1.25	-4.04 – 1.53	0.376
Asian	**0.13**	**0.02 – 0.45**	**<0.01**	**0.31**	**0.10 – 0.80**	**<0.05**	-3.83	-7.90 – 0.24	0.065
Other	0.7	0.21 – 2.02	0.528	0.44	0.12 – 1.31	0.164	-3.23	-8.29 – 1.83	0.21
**Health Insurance**									
Private	1.0	---	---	1.0	---	---	---	---	---
Medicaid	0.64	0.27 – 1.45	0.304	0.74	0.32 – 1.62	0.459	0.73	-3.09 – 4.56	0.707
Medicare	0.72	0.39 – 1.30	0.284	0.75	0.41 – 1.33	0.325	1.76	-1.04 – 4.56	0.216
No Insurance	0.65	0.24 – 1.60	0.364	0.67	0.26 – 1.62	0.389	-0.02	-4.29 – 4.24	0.992
**Education Completed**									
Bachelor/ Associate	1.0	---	---	1.0	---	---	---	---	---
Graduate Degree	0.62	0.29 – 1.26	0.195	0.8	0.40 – 1.58	0.524	-1.11	-4.44 – 2.23	0.515

High School or Less	0.85	0.50 – 1.45	0.554	0.75	0.44 – 1.27	0.286	-1.42	-3.98 – 1.14	0.276
**Employment Status**									
Full-Time	1.0	---	---	1.0	---	---	---	---	---
Not Employed	**0.51**	**0.30 – 0.87**	**<0.01**	**0.55**	**0.32 – 0.92**	**<0.05**	0	-2.52 – 2.52	1
Part-Time	**0.41**	**0.18 – 0.87**	**<0.05**	**0.39**	**0.18 – 0.82**	**<0.05**	-0.72	-4.15 – 2.71	0.679

	**Tested Positive for SARS-CoV-2 (Yes/No)**			**Experienced COVID-19 Symptoms (Yes/No)**			**Days Experienced COVID-19 Symptoms**		
**Indep. Variable**	**Odds Ratio (Unadj)**	**95% CI**	**p-Value**	**Odds Ratio (Unadj)**	**95% CI**	**p-Value**	***ß Coefficient***	**95% CI**	**p-Value**
**Underlying Medical Conditions**									
No	1.0	---	---	1.0	---	---	---	---	---
Yes	**3.3**	**1.99 – 5.57**	**<0.001**	**5.07**	**3.04 – 8.62**	**<0.001**	1.7	-0.61 – 4.01	0.148
**Diabetes**									
No	1.0	---	---	1.0	---	---	---	---	---
Yes	**2.88**	**1.60 – 5.25**	**<0.001**	**3.93**	**2.16 – 7.34**	**<0.001**	0.99	-1.88 – 3.85	0.498

**Hypertension**									
No	1.0	---	---	1.0	---	---	---	---	---
Yes	**2.66**	**1.59 – 4.49**	**<0.001**	**3.96**	**2.35 – 6.75**	**<0.001**	**2.71**	**0.25 – 5.16**	**<0.05**
**Dyslipidemia**									
No	1.0	---	---	1.0	---	---	---	---	---
Yes	1.85	1.00 – 3.42	0.051	**2.17**	**1.18 – 4.04**	**<0.05**	0.24	-2.77 – 3.25	0.875
**Vitamin D Supplementation**									
No	1.0	---	---	1.0	---	---	---	---	---
Yes	**0.15**	**0.07 – 0.28**	**<0.001**	**0.11**	**0.06 – 0.22**	**<0.001**	**-4.66**	**-7.01 – -2.30**	**<0.001**
**Received COVID-19 Vaccine**									
No	1.0	---	---	1.0	---	---	---	---	---
Yes	**0.58**	**0.35 – 0.95**	**<0.05**	**0.58**	**0.36 – 0.94**	**<0.05**	**-3.27**	**-5.56 – -0.99**	**<0.05**
**Exposed to Omicron or Delta Strain**									
Delta	1.0	---	---	1.0	---	---	---	---	---
Omicron	**1.79**	**1.09 – 2.94**	**<0.05**	**0.38**	**0.23 – 0.61**	**<0.001**	**-2.56**	**-4.88 – -0.24**	**<0.001**
**Previously Received Oral Polio Vaccine**									

Yes	1.0	---	---	1.0	---	---	---	---	---
No	**3.92**	**2.27 – 6.88**	**<0.001**	**4.06**	**2.35 – 7.17**	**<0.001**	**6.53**	**4.03 – 9.04**	**<0.001**
**Vitamin D x Education**									
(ref)	1.0	---	---	1.0	---	---	---	---	---
Vitamin D (Yes)	**0.1**	**0.03 – 0.28**	**<0.001**	**0.07**	**0.02 – 0.21**	**<0.001**	--	--	--
Education Completed (Graduate Degree)	**0.38**	**0.15 – 0.91**	**<0.05**	0.48	0.20 – 1.12	0.093	--	--	--
Education Completed (High School or Less)	0.8	0.42 – 1.52	0.492	0.68	0.35 – 1.31	0.252	--	--	--
Yes x Graduate Degree	**6.38**	**1.08 – 38.50**	**<0.05**	**6.79**	**1.25 – 38.83**	**<0.05**	--	--	--
Yes x High School or Less	1.07	0.21 – 5.46	0.938	0.91	0.15 – 5.00	0.914	--	--	--
